# Digital Insights into Workplace Breastfeeding in Indonesia: A Google Trends Analysis of Barriers and Opportunities

**DOI:** 10.3390/nu17213433

**Published:** 2025-10-31

**Authors:** Ray Wagiu Basrowi, Tonny Sundjaya, Dessy Pratiwi, Nurfadilah M. Rajab, Rachel Amanda, Heru Komarudin, Gassani Amalia

**Affiliations:** 1 Occupational Medicine Division, Department of Community Medicine, Faculty of Medicine, Universitas Indonesia, Salemba Raya 6, Central Jakarta 10430, Indonesia; 2 Indonesia Health Development Center, Percetakan Negara 29, Central Jakarta 10560, Indonesia; s_ton77@yahoo.com (T.S.); pratiwi.dessy@gmail.com (D.P.); fadilahmrajab@gmail.com (N.M.R.); rachelamanda0308@gmail.com (R.A.); 3 Health Collaborative Center, TB Simatupang 18C, South Jakarta 12430, Indonesia; 4 Department of Epidemiology, Faculty of Public Health, Universitas Indonesia, Depok 16424, Indonesia; 5 BUMN Foundation, Teuku Cik Ditiro 27A, Central Jakarta 10310, Indonesia; herrukomarudin30@gmail.com; 6 Varians Health Statistic, Cipunagara Raya 4, South Tangerang 15411, Indonesia; gassani.amalia@hotmail.com

**Keywords:** breastfeeding, breastfeeding support, breastfeeding campaign, workplace, Google Trends

## Abstract

**Background/Objectives**: Exclusive breastfeeding rates in Indonesia remain low, particularly among working mothers, despite government policies and the substantial contribution of women to the national economy. Inadequate workplace support, with only 21.5% of working mothers having access to proper lactation facilities, is a key barrier. This study aimed to analyze Google Trends search data to understand the barriers and opportunities regarding workplace breastfeeding support in Indonesia, providing a data-driven foundation for advocacy campaigns and policy development. **Methods**: We conducted a retrospective analysis of Google Trends data from July 2020 to July 2025. Temporal and geographic search patterns for selected keywords, along with related queries and topics, were analyzed using a normalized relative search volume index (0–100). **Results**: “Lactation room” was the dominant, foundational search term with sporadic, event-driven peaks. Search interest in “exclusive breastfeeding” was consistently high (“evergreen”), while “World Breastfeeding Week” showed predictable seasonal peaks. Geographically, the need for basic infrastructure was nationally distributed, but searches for practical solutions, deeper topics, and event momentum were concentrated in urban economic centers. A nationwide knowledge gap on rights was identified. Analysis of “Rising Queries” and “Topics” revealed a shift in user focus from general information toward specific needs regarding rights, policy, and community support. **Conclusions**: The geographic and temporal alignment of user-identified needs with campaign momentum validates a targeted, multi-layered advocacy strategy. A three-pillar approach combining data-driven communication, workplace policy change, and multi-sectoral collaboration is recommended to improve breastfeeding support for working mothers in Indonesia.

## 1. Introduction

Breastfeeding and human milk represent the normative standard for infant nutrition, promoting optimal short- and long-term nutritional and neurodevelopmental outcomes. Studies reported that 6 months exclusive breastfeeding associated with lowering risk of neonatal and infant mortality, lower respiratory tract infection, severe or persistent diarrhea, otitis media, asthma, eczema, colitis, childhood obesity, diabetes, and leukaemia [1,2]. Breastfeeding also benefits mothers which it become natural contraception, lowering risk of obesity, non-communicable disease, and post-natal depression [3]. The World Health Organization (WHO) and the United Nations International Children’s Emergency Fund (UNICEF) recommends exclusive breastfeeding for the first six months of life, followed by continued breastfeeding with appropriate complementary foods for up to two years or beyond [4]. Despite these benefits and global recommendations, exclusive breastfeeding prevalence was approximately 48% in 2023, with rates in Indonesia ranging from 51.7% to 69.7% [4,5,6,7].

With the increasing participation of Indonesian women in the labor force and their contribution to national productivity [8,9], adequate workplace support is crucial to prevent breastfeeding failure or premature weaning [10]. Efforts have been in place to support breastfeeding at workplace in Indonesia, including educational campaigns through broadcast and social media, as well as national policies that oblige employers to support exclusive breastfeeding, including technical guidance to provide dedicated lactation rooms [3]. An Indonesian study showed workplace lactation facilities availability increased exclusive breastfeeding practice nearly threefold [11].

However, despite these efforts and benefits, a gap in implementation and practice of breastfeeding among working mothers persists. An estimated 45% of mothers in Indonesia stopped breastfeeding at three months postpartum, which affected by Indonesian Labor Law Number 13 Year 2013 Article 82 that limits the maternity leave to only 3 months [6,11]. Moreover, the prevalence of breastfeeding among working mothers in Indonesia remains low, for instance, rates among factory workers were as low as 19% in 2015, far below the national average of 32% at the time [11]. This disparity is likely influenced by inadequate workplace support, as only 21.5% of working mothers have access to proper lactation facilities and just 7.5% have access to an adequate lactation program [11]. Another study found that 15% of white-collar works and 17% of blue-collar workers never breastfed nor expressed milk at work [12].

With Indonesia’s internet penetration rate reaching 80.66% in 2025, which corresponds to 229 million users, the digital landscape is deeply integrated into daily life [13]. Given this high level of digital engagement and Google Search’s market dominance, Google Trends data offers valuable insights into population health interests [14,15], as it provides large-scale, unfiltered data on public information-seeking behavior through analyses of billions of daily searches to provide data on geospatial and temporal patterns. While Google Trends has been widely applied in health research, its use to investigate workplace breastfeeding support in Indonesia remains unexplored. Therefore, this study aimed to analyze Google Trends search patterns to identify public information needs and topics of interest regarding the barriers and opportunities for workplace breastfeeding support in Indonesia. The objective was to provide a data-driven foundation for developing digital campaigns, policy advocacy, and content that can directly impact corporate ecosystems and working mothers.

## 2. Materials and Methods

This study utilized Google Trends to analyze temporal patterns, geographic distributions, related queries, and associated topics for keywords concerning workplace breastfeeding support in Indonesia. Data from five years (July 2020–July 2025) were extracted across all Google platforms (including Web, Image, News, YouTube, and Google Shopping) to identify both seasonal patterns and long-term trends. The tool aggregates historical search logs and calculates the proportion of queries for a specified term relative to all searches within a chosen time and region. This relative search volume is then normalized and presented on a scale of 0–100, where 100 signifies the peak search interest for that term during the period [14].

Table 1 details the keywords used for the analysis, which were selected based on a PubMed search for terms related to workplace breastfeeding support [10,16]. Keyword selection was conducted through a two-stage process. First, a literature review was performed on PubMed, Google Scholar, grey literature review, and institutional reports on studies from year 2015 to 2025 to identify core concepts related to workplace breastfeeding support. Second, a panel of two senior researchers (R.W.B., T.S.) with expertise in public health and occupational medicine reviewed and finalized the keyword list, translating them into Indonesian where appropriate to capture local search behavior. The keywords were then categorized as ‘Primary’ to reflect core barriers and solutions, and ‘Secondary’ to provide context on general interest and awareness events (Table 1). “Primary” keywords were used for the main analysis, while “Secondary” keywords provided context by relating annual awareness events to the audience’s continuous information needs. A geographical analysis was performed to map the distribution of search interest across all Indonesian provinces, identifying regions with the highest query volumes. Furthermore, an analysis of “Related Queries” was conducted to identify both top (popular) and rising (trending) searches to understand user intent better.

## 3. Results

### 3.1. Time Trend Analysis

**Figure 1:**
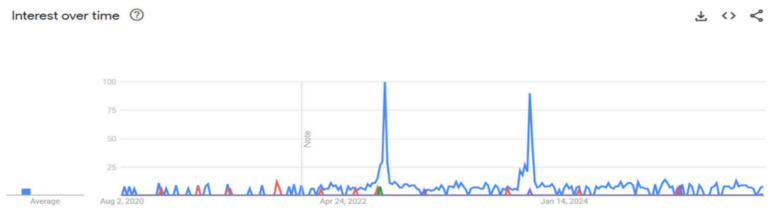
Time-trend analysis of primary keywords related to workplace breastfeeding support (2020–2025). The *y*-axis represents the relative search interest on a normalised scale of 0–100 based on “Search Interest Index”. The blue line denotes “lactation room,” red denotes “breastfeeding for working mothers,” green denotes “rights of breastfeeding mothers at the office,” and purple denotes “portable breast pump.” The search term “managing expressed breast milk at the office” (yellow) exhibited negligible search volume and is not visible.

As shown in Figure 1, the primary keyword analysis revealed a clear dominance of the search term “lactation room”. Its search interest was consistently higher than all other primary keywords and was characterised by exceptionally high peaks. Also, this primary analysis revealed distinct temporal patterns between keywords. The search term “Pekan ASI Sedunia” (World Breastfeeding Week) exhibited a seasonal pattern, with sharp, brief peaks occurring annually in early August. In contrast, interest in “lactation room” was sporadic. Two exceptionally high and sharp peaks for “lactation room” were identified around the end of the second quarter of 2022 and in early 2024. In contrast, more specific keywords such as “rights of breastfeeding mothers at the office”, “expressed breast milk management at the office”, and “breastfeeding for working mothers” demonstrated a comparatively low and consistently flat search volume throughout the entire search period.

**Figure 2:**
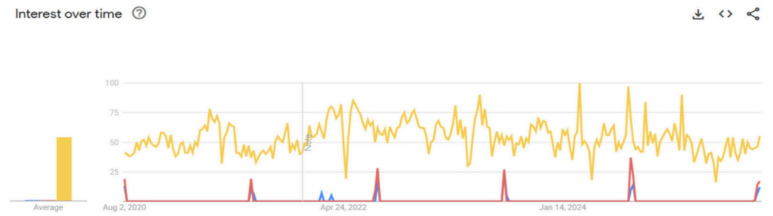
Time-trend analysis of secondary keywords related to breastfeeding (2020–2025). The *y*-axis represents the relative search interest on a normalised scale of 0–100 based on “Search Interest Index”. The graph illustrates “exclusive breastfeeding” (yellow line), “Pekan ASI Sedunia” (red line) and “World Breastfeeding Week” (blue line).

In contrast to the pattern in Figure 1, Figure 2 reveals two distinct temporal patterns in the secondary keyword analysis. The search term “exclusive breastfeeding” demonstrated a consistently high search volume throughout the five-year period, with normal fluctuations. In contrast, both “Pekan ASI Sedunia” and its English equivalent, “World Breastfeeding Week,” exhibited a baseline of low search activity punctuated by sharp, brief, and predictable peaks each year in early August. While the search volume for the English term was generally lower, its peak pattern followed the same annual trend.

### 3.2. Geographic Analysis

The geographic analysis of search data revealed several contrasting patterns of public interest across Indonesia. Search interest for basic infrastructure, such as “lactation room”, was consistently high and evenly distributed. In contrast, interest in practical solutions and specific health topics was highly concentrated in urban hubs. The highest search volumes for “exclusive breastfeeding” and “portable breast pump” originated from economic centres, including DKI Jakarta, Banten, and DI Yogyakarta. Similarly, engagement with annual events like “Pekan ASI Sedunia” was primarily found in these urban provinces. Finally, the analysis suggested a potential gap in public awareness regarding policy, as search interest for terms such as “rights of breastfeeding mothers at the office” was uniformly low across the entire country.

### 3.3. Related Queries

An analysis of related user searches revealed two distinct categories of intent: Top Queries and Rising Queries, as detailed in Table 2. Top Queries were generally broad and informational, focusing on the annual awareness event (e.g., “World Breastfeeding Week theme”, “logo”) and foundational knowledge (“importance of exclusive breastfeeding”). In contrast, Rising Queries reflected specific, action-oriented needs directly related to the barriers faced by working mothers. These included searches for practical logistics (“method of expressed breastmilk storage”), policy and rights (“maternal leave policy”, “breastfeeding mothers’ right in workplace”), and support systems (“spouse support”, “online breastfeeding consultation”), as well as the community-specific term “Pejuang ASI” (a popular local term for breastfeeding advocates or ‘fighters’).

### 3.4. Related Topics

The analysis of related topics, summarised in Table 3, provides a broader context of user interest categories, revealing both established “Top Topics” and emergent “Rising Topics”.

The analysis of “Related Queries” and “Related Topics” utilized the standard output generated by the Google Trends platform. “Top Queries” represent queries with the highest search volume during the specified period, while “Rising Queries” are those with the most significant growth in search frequency. Similarly, “Related Topics” groups user searches into conceptual categories. The data analysis for this study was descriptive, involving visual inspection of time-series graphs to identify patterns (e.g., seasonality, peaks) and a thematic analysis of the related queries and topics to understand user intent and interest.

## 4. Discussion

Supporting breastfeeding in the workplace is not just a health imperative but also a critical component of sustainable development. While we recognize that search trends are a complex signal influenced by the broader digital ecosystem, our analysis of this data, interpreted as a proxy for public information-seeking behavior, provides a foundation for developing such a sustainable strategy. Ensuring mothers can continue breastfeeding after returning to work directly contributes to several Sustainable Development Goals (SDGs), including SDG 3 (Good Health and Well-being), SDG 5 (Gender Equality), SDG 8 (Decent Work and Economic Growth), and SDG 10 (Reduced Inequalities) [17].

Our analysis of Google Trends data provides a foundation for developing such a sustainable strategy. The primary keyword analysis revealed that “lactation room” functions as the foundational search term for workplace breastfeeding support in Indonesia. Its search volume was consistently higher than that of other primary keywords. It was characterized by sporadic, event-driven peaks, suggesting it is the main entry point for users seeking information on this topic. The user’s search journey likely begins with this general term before progressing to more specific queries such as “rights of breastfeeding mothers at the office” or “managing expressed breast milk at the office”. Although these more detailed search terms showed consistently lower volumes, this does not diminish their importance; it merely clarifies the user’s path to information. The anomalous nature of the peaks for “lactation room” suggests they were triggered by external events, such as viral news stories, new regulations, or large-scale advocacy campaigns.

The secondary keyword analysis uncovered two distinct user behaviors that inform a dual approach to a sustainable advocacy strategy. The search term “exclusive breastfeeding” demonstrated a high, stable search volume, highlighting its role as a fundamental and “evergreen” concept with a persistent, ongoing information need among new and expectant parents. This finding is the basis for a Foundation Strategy, which aims to meet this continuous demand by creating a sustainable, year-round resource hub to build long-term authority and consistently reach the target audience. In contrast, “Pekan ASI Sedunia” (World Breastfeeding Week) functioned as a powerful annual catalyst, exhibiting low baseline interest but experiencing sharp, predictable peaks in August that concentrate public attention on the issue. This informs the Momentum Strategy, which leverages this annua event to launch high-impact campaigns and new messages, and activations to maximize public engagement in a short period.

Geographic analysis revealed three contrasting patterns of interest. First, the need for basic infrastructure like a “lactation room” was a topic of widespread search interest with relatively even distribution across all provinces. Second, deeper topics such as “exclusive breastfeeding”, practical solutions such as “portable breast pump”, and event momentum from “Pekan ASI Sedunia” were highly concentrated in urbanized, economically active provinces, especially on Java Island. Finally, interest in policy-related terms such as “rights of breastfeeding mothers” was uniformly low across Indonesia, suggesting a potential gap in public awareness. However, this pattern may also be significantly influenced by socioeconomic factors and the digital divide, where populations in less-developed regions may have less access to the internet or lower digital literacy to formulate such specific, policy-related queries [18]. Therefore, designing equitable educational programs requires strategies that account for these digital access disparities.

The analysis of related queries and topics provided more profound insights into the true voice of the target audience. “Top Queries” were broad and informational, indicating a wide entry point for the audience, while “Rising Queries” represented the authentic voice of working mothers, reflecting their specific, unresolved barriers related to rights and facilities at workplaces (breastfeeding rights, lactation rooms), practical logistics (breastmilk storage), and the need for support systems (spouses and online consultations). Similarly, “Top Topics” framed the issue within the larger contexts of “Maternal and Child Health” and “Parenting,” while “Rising Topics” showed a critical shift in perspective—from viewing support as corporate goodwill to a “fundamental worker’s right”. This shift was complemented by rising interest in mental health, online communities, and supporting products.

The findings from this study present an opportunity to develop targeted, multi-layered strategy to promote breastfeeding in the workplace, including content that directly addresses working mother’s real concerns. We propose an overall strategy structured into three synergistic pillars to promote breastfeeding in the workplace.

### 4.1. Pillar 1: Data-Driven Communication and Digital Marketing

Given that a majority of Indonesian adults have been exposed to social media or broadcast media, these platforms are powerful tools for health promotion, serving as a key communication channel for the new generation of mothers whose cultural attitudes are heavily influenced by peer interactions online [19,20,21]. This first pillar involves creating segmented digital content based on our query analysis to address specific user needs. Evidence shows that such campaigns can improve breastfeeding knowledge, adherence, self-efficacy, perceived social support, initiation and continuation of breastfeeding [19,20,22,23]. This promotion is beyond behavioral change, as breastfeeding health promotion is also linked to infant health benefits, including reduced odds of respiratory illness and diarrhea, also related to better linear growth [24].

To be effective, this communication strategy should employ a dual approach: a national awareness campaign focused on basic infrastructure, coupled with intensive, targeted investment (e.g., paid advertising, billboards) in economic centers [19,25]. To maximize the impact of events like World Breastfeeding Week, a pre-campaign phase should begin several months prior to build momentum, and advocacy should be sustained year-round [26]. The geographic and temporal alignment of user-identified needs (evidenced by high search volumes for “exclusive breastfeeding” in economic centers) with campaign momentum (from “Pekan ASI Sedunia”) signifies that a data-driven campaign message can inherently reach its most receptive audience.

However, communication-only campaigns face barriers, such as ensuring adequate message exposure, as low media frequency can be insufficient [22,27,28]. Therefore, these efforts must be complemented by the initiatives in the other pillars.

### 4.2. Pillar 2: Comprehensive Workplace Lactation Promotion Model

This pillar focuses on translating data into a holistic and empowered support for working mothers. Evidence clearly shows that longer maternity leave and positive workplace interventions, such as dedicated nursing breaks and lactation rooms, are associated with better breastfeeding rates [16,20,29]. Advocacy efforts should focus on ensuring the equitable coverage and enforcement of existing legislation. Crucially, maternity protection should be extended to women working in the informal economy, a key sector that employs the majority of women in many developing regions [30]. In the long term, the findings from this study can be framed as a compelling business case for corporations, linking breastfeeding support to benefits such as improved talent retention and providing a basis for launching workplace certification programs [31]. Overcoming potential employer resistance and funding constraints requires framing this support not as a cost, but as a strategic investment in human capital [32]. Advocacy efforts can be strengthened by launching targeted pilot programs in key corporations to generate local data on the positive return on investment, such as reduced absenteeism and higher loyalty, thereby creating a compelling, evidence-based case for wider adoption and overcoming legislative inertia [33].

This model is built on four key components [34,35,36]. Firstly, comprehensive policy and regulation which covers longer maternity leave until 6 months postpartum, flexible working arrangements post-return, nursing breaks for daily breaks, limitation of extra or out-of-town work for breastfeeding employees. Secondly, a supportive physical environment is crucial to improve the breastfeeding rates, including lactation room that equipped with comprehensive supplies (refrigerator, chair, sink, pump, sterilizer, and water dispenser). Thirdly, education through blended method such as lecturers, discussion, private counselling, and digital engagement is key to female employees as well as male employees whose wives are pregnant. Ultimately, the success of this model require commitment and coordinated effort across peers- and managerial-level [34,35,36,37].

### 4.3. Pillar 3: Supporting Innovation and Multi-Sectoral Collaboration

The final pillar aims to build a supportive ecosystem through service innovation and broad partnerships. The limitations of communication-only campaigns highlight the need for a blended approach complemented by community-based programs, such as interpersonal communication and support groups. This leads to opportunities for Supporting Product and Service Innovation, such as developing B2B corporate lactation counseling services to meet the high demand identified in our “Rising Queries” analysis. Effective community-level strategies include providing prenatal and postnatal counseling, lay and professional support, and targeted education [38,39]. A key element of this support is equipping mothers, healthcare providers, and communities with the knowledge to optimal nutritional status of mothers and adequate preparation before returning to work, debunk misconceptions about commercial milk formula equivalence, and to understand unsettled baby behaviors as a normal phase of development [40,41]. It is also crucial to educate mothers on addressing or preventing self-reported insufficient milk to avert the use of prelacteal feeds, which are a major risk factor for the premature termination of breastfeeding [40].

Ultimately, these individual and community efforts must be reinforced by a strong policy framework and multi-sectoral collaboration [30]. Successful initiatives in other developing countries demonstrate the importance of partnerships between government (across health, labor, and education sectors), international organizations, NGOs, and academia [40,42]. In Indonesia setting, this would involve collaboration between the Ministry of Health (for clinical guidelines), the Ministry of Manpower (for policy enforcement and workplace inspection), private sector associations (to encourage corporate adoption), and non-governmental organizations (for community-level advocacy). Such collaborations are essential for driving the evidence-based advocacy and national policies needed to protect and promote breastfeeding for all mothers.

### 4.4. Strengths and Limitations

To our knowledge, this is the first study to use Google Trends data to develop an actionable strategic framework for workplace breastfeeding support in Indonesia. Its primary strength lies in using real-time, revealed population search data to provide an unfiltered view of public information needs on this critical health issue. However, the interpretation of search volume as a direct proxy for public concern must be approached with caution, as search behavior is a complex signal influenced by multiple external factors.

First, search trends can be heavily influenced by exogenous events such as media coverage or policy announcements. An anomalous peak in searches for “lactation room”, for example, may reflect a prominent news story rather than a sudden change in the underlying need for facilities. While we posit that these events create valuable “windows of opportunity” for advocacy, it is methodologically crucial to distinguish these transient spikes from the stable, “evergreen” search patterns (e.g., “exclusive breastfeeding”) that signal chronic information gaps.

Second, the data is subject to algorithmic influences and the digital divide. Our sample is inherently limited to the segment of the population with internet access and the digital literacy to articulate their needs as search queries. This is particularly relevant in the Indonesian context, where significant disparities in internet penetration and digital skills persist between urban centers like Jakarta and more rural, remote islands [43]. Consequently, the finding that complex searches are concentrated in urban economic centers likely reflects not only a higher prevalence of formal sector employment but also this underlying socioeconomic sampling bias. This means the perspectives of rural, less digitally connected, or lower-income working mothers are likely underrepresented in this dataset, a common limitation in digital health surveillance studies [44].

Finally, while this study identifies powerful trends, future research should build upon this foundation. While visual analysis of seasonality is a common starting point in Google Trends research, future work should validate these findings with quantitative seasonality assessment. Furthermore, this study does not statistically correlate search trends with real-world outcomes, a powerful application of Google Trends data seen in other studies [15,45]. a key future direction is to integrate our infoveillance data with real-world public health outcomes. We propose a subsequent longitudinal study to conduct a time-series analysis (e.g., using ARIMA or vector autoregression models) correlating the identified search trends with key metrics such as national exclusive breastfeeding rates (e.g., from Indonesia’s National Health Survey) and workplace policy adoption (e.g., a newly established registry of companies with certified lactation facilities) [16,46,47]. Such an analysis would allow us to model the temporal relationship between public interest and policy action, for example, testing whether search spikes for “lactation room” precede increases in policy implementation, or if campaign momentum (tracked via “Pekan ASI Sedunia”) correlates with subsequent improvements in breastfeeding continuation rates [15]. This econometric approach would provide a more robust evidence base to strengthen advocacy arguments.

To overcome the limitations of digital-only data, which can be decontextualized, a mixed-methods approach is essential to provide a richer evidence base for the proposed strategies [48,49]. Future research should use qualitative methods to validate and contextualize our findings. For instance, semi-structured interviews with working mothers could confirm whether ‘lactation room’ is indeed the foundational entry point in their information-seeking journey and uncover nuanced barriers, such as perceived managerial support or workplace culture, that search data cannot capture [50]. Furthermore, as suggested by the reviewer, interviews with corporate stakeholders (e.g., HR managers) would be crucial to identify supply-side barriers to infrastructure prioritization and inform the sequencing of advocacy efforts.

## 5. Conclusions

By analyzing five years of Google Trends data, we identified distinct user behaviors and needs, barriers and opportunities for workplace breastfeeding support in Indonesia. The foundational search interest in “lactation room” reveals the public’s primary concern for basic infrastructure, while the evergreen interest in “exclusive breastfeeding” signals a continuous, unmet need for information. In contrast, predictable seasonal peaks around World Breastfeeding Week show clear opportunities for targeted campaigns. Geographic analysis revealed that while the need for facilities is national, searches for practical solutions are concentrated in urban economic centers, suggesting a potential knowledge gap in policy and rights, particularly outside urban centers. The alignment of user-identified needs with campaign momentum in these key urban areas validates a targeted, multi-layered advocacy strategy. Therefore, we recommend a three-pillar approach combining data-driven communication, a comprehensive workplace lactation promotion model, and multi-sectoral collaboration. This data-driven strategy offers a promising roadmap to create sustainable support systems that empower working mothers and contribute to national health and economic goals.

## Figures and Tables

**Table 1:** Keyword classification based on relevance and search type.

No.	Keyword	Language	Relevance	Type
1	“Lactation room”	Indonesian	Solution/Infrastructure	Primary
2	“Breastfeeding for working mothers”	Indonesian	Barriers/Problem	Primary
3	“Managing expressed breast milk at the office”	Indonesian	Technical & practical information	Primary
4	“Rights of breastfeeding mothers at the office”	Indonesian	Advocacy & social issues	Primary
5	“Portable breast pump”	Indonesian	Supporting product	Primary
6	“World Breastfeeding Week”	English	Global awareness event	Secondary
7	“*Pekan ASI Sedunia*”	Indonesian	Local term	Secondary
8	“Exclusive Breastfeeding”	Indonesian	The ultimate goal to be achieved	Secondary

**Table 2:** Top and rising Google search queries related to breastfeeding.

Top Queries	Rising Queries
“World Breastfeeding Week theme [11]” “World Breastfeeding Week logo” “World Breastfeeding Week poster” “Breastmilk benefit for infants” “Breastmilk benefit for mothers” “Importance of exclusive breastfeeding”	“Breastfeeding mothers’ right in workplace” “Lactation room at working place or public facility” “Method of expressed breastmilk storage” “Maternal leave policy in Indonesia” “Spouse support during breastfeeding” “Online breastfeeding consultation” “Pejuang ASI”

**Table 3:** Top and rising Google search topics related to breastfeeding.

Top Topics	Rising Topics
“Maternal and child health” “Parenting” “Company management and policy”	“Human rights or worker rights” “Regional government or public policy” “Psychology or mental health” “Online community or discussion forum” “Supporting product for working mothers”

## Data Availability

The data are available from the corresponding author upon reasonable request due to privacy restrictions.
